# Research Tools for the Functional Genomics of Plant miRNAs During Zygotic and Somatic Embryogenesis

**DOI:** 10.3390/ijms21144969

**Published:** 2020-07-14

**Authors:** Anna Maria Wójcik

**Affiliations:** University of Silesia in Katowice, Faculty of Natural Sciences, Institute of Biology, Biotechnology and Environmental Protection, Jagiellonska 28, 40-032 Katowice, Poland; anna.wojcik@us.edu.pl; Tel.: +48-322-009-428

**Keywords:** miRNA, *MIRNA* genes, MIM, STTM, amiRNA, in situ hybridisation, miRNA-resistance, functional genomics, embryogenesis, plant

## Abstract

During early plant embryogenesis, some of the most fundamental decisions on fate and identity are taken making it a fascinating process to study. It is no surprise that higher plant embryogenesis was intensively analysed during the last century, while somatic embryogenesis is probably the most studied regeneration model. Encoded by the *MIRNA*, short, single-stranded, non-coding miRNAs, are commonly present in all Eukaryotic genomes and are involved in the regulation of the gene expression during the essential developmental processes such as plant morphogenesis, hormone signaling, and developmental phase transition. During the last few years dedicated to miRNAs, analytical methods and tools have been developed, which have afforded new opportunities in functional analyses of plant miRNAs, including (i) databases for in silico analysis; (ii) miRNAs detection and expression approaches; (iii) reporter and sensor lines for a spatio-temporal analysis of the miRNA-target interactions; (iv) in situ hybridisation protocols; (v) artificial miRNAs; (vi) *MIM* and *STTM* lines to inhibit miRNA activity, and (vii) the target genes resistant to miRNA. Here, we attempted to summarise the toolbox for functional analysis of miRNAs during plant embryogenesis. In addition to characterising the described tools/methods, examples of the applications have been presented.

## 1. Plant miRNAs, What We Are Dealing With?

**Figure 1:**
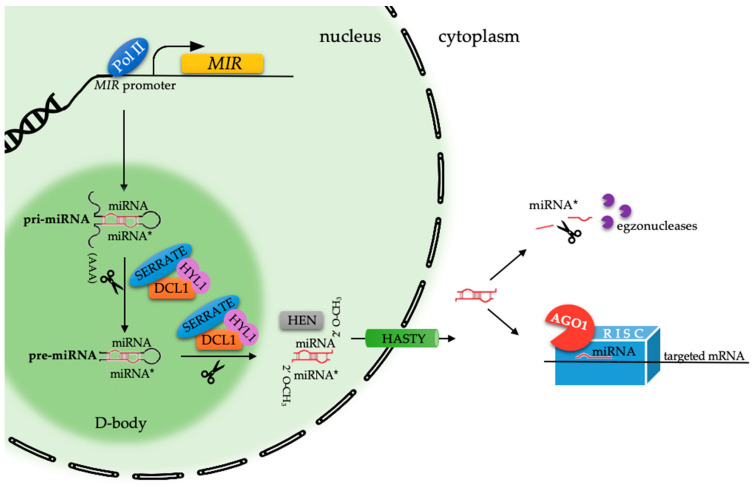
A schematic overview of the plant microRNA (miRNA) biogenesis pathway. Pol II: DNA-dependent RNA polymerase II; *MIR*: *MIRNA* gene; pri-miRNA: Primary-miRNA; pre-miRNA: Precursor-miRNA; DCL1: DICER-like1; HYL: HYPONASTY LEAVES1; SERRATE; HEN1: HUA ENHANCER1; 2′O-CH3: Methylated 2′ hydroxyl group; HASTY; AGO: ARGONAUTE; RISC: RNA Induced Complex; D-body: Dicing-body.

For many years, knowledge about the RNA functions has been limited to one type of protein-coding RNA, mRNA (messenger RNA), and two non-coding RNAs—rRNA (ribosomal RNA), which builds the ribosomes, and tRNA (transfer RNA), which is responsible for the transport of amino acids. A big step forward was when the non-coding RNAs (ncRNA) that are engaged in the regulation of gene expression were described. The division into infrastructural ncRNA and regulatory ncRNA has also been performed. Included in the group of infrastructural ncRNA are rRNA, tRNA, small nuclear RNA (snRNA), and small nucleolar RNA (snoRNA). Meanwhile, in the group of regulatory ncRNA, several types have been distinguished as micro RNA (miRNA), PIWI-interacting RNA (piRNA; animals only), small interfering RNA (siRNA), promoter-associated RNA (PAR), enhancer RNA (eRNA), and long non-coding RNA [1,2,3,4]. The regulatory ncRNA groups differ in the lengths of their molecules, transcript origin, and biogenesis pathway components. Among the ncRNAs, the most interest has been focused on the functions of two groups of small RNAs—miRNA and siRNA [3]. miRNAs, which are a class of tiny, around 21 nucleotides, endogenous ncRNAs were described for the first time in 1993 in nematode *Caenorhabditis elegans* [5] and in plants in 2002 in Arabidopsis [6]. The miRbase in 2002 had information about 218 miRNA loci in five plant species. Currently, the presence of miRNAs has been proven in the entire Plantae starting from green algae, ferns, and mono-, di-cotyledons showing a strong evolutionary conservatism [7]. Today, we can find more than 10,200 records for mature miRNA molecules in plants from 92 species in the miRbase, while for Arabidopsis, there is information about 428 mature miRNA molecules (http://www.mirbase.org; 04.2020 [8]). miRNAs are encoded by *MIRNAs* (*MIRs*), which are intergenic or intronic genes that are present in plant genomes in one hundred to as many as several hundred loci, which can be located in genomic regions that are distinct from the known transcription units and that have their own promoter and terminator sequences such as mono or polycistronic *MIRs*. In turn, intronic *MIR*s are processed from the introns of the protein-coding genes [9,10]. There are more than 2000 miRNA families in the most recent 22.1 release of miRbase [8], which reveal that the largest *MIR* families can have more than sixty members (miR2592 in *Medicago trancatula*) that are coding similar, almost identical mature miRNA molecules (http://www.mirbase.org). The multistep process of miRNA biogenesis (Figure 1) initiates the transcription of the primary miRNAs (pri-miRNA) structures by DNA-dependent RNA polymerase II (Pol II), which recognises the TATA box in the *MIR* promoter sequences. The pri-miRNAs containing both the 5′cap and 3′poly A tail are processed into one strand precursor miRNA (pre-miRNA) molecules by DICER-like1 RNase III endonuclease (DCL1), which catalyses the production of most plant miRNAs. In Arabidopsis, four different DCL proteins have been described and all are engaged in a specific sRNA-dependent gene silencing pathway with some redundancy [11]. The pre-miRNA is further processed by DCL1 along with the double-stranded RNA-binding protein HYPONASTY LEAVES1 (HYL1) and the zinc-finger protein SERRATE to produce a 21-nt miRNA/miRNA* (passenger strand designated with asterisk) duplex inside the nucleus within specialised compartments, which are called Dicing-bodies. The miRNA/miRNA* duplex is then methylated at the 3′ terminal hydroxyl group (2′OH) by the HUA ENHANCER1 (HEN1), which is a small RNA methyltransferase that acts in a sequence-independent and structure-dependent manner. The 2′OH methylation is crucial for protecting an unwound miRNA molecule from small RNA-degrading nucleases. The methylated miRNA/miRNA* duplex is thought to be transported to the cytoplasm by HASTY. Only one guide strand of the miRNA/miRNA* duplex (miRNA) is selectively loaded into the RNA-induced silencing complex (RISC) via binding with the ARGONAUTE (AGO) protein, while the passenger strand (miRNA*) is degraded. The selection of the guide strand is dependent on the 5′ end nucleotide, which is also important in the AGO binding step. Most plant miRNAs carry the 5′ terminal U (uridine), which is usually bound by AGO1 RNaseH-like that has an endonuclease activity; it is one of the ten AGO proteins that are found in Arabidopsis and as many twenty-two in *Glycine max* [12]. The loaded mature miRNA in the RISC regulates the target genes mainly at the post-transcriptional (PTGS) level in two different ways, which results in transcript cleavage or translation repression. The miRNA-RISC mode of action is dependent on the degree of the sequence complementarity between the miRNA and their target. Although a high degree of sequence complementarity is required for mRNA cleavage, there are examples such as miR172, miR171, and miR156, which may regulate the targets *APETALA2*, *SCARECROW-LIKE PROTEIN4,* and *SQUAMOSA PROMOTER BINDING PROTEIN-LIKE3* (*SPL3*)*,* respectively, by both the mechanisms of transcript cleavage and translation repression indicating that this is not everything. Lastly, it has been found that the bond between a target transcript and a ribozyme may trigger the translational repression as a miRNA mode of action even for an miRNA-target pair with a high degree complementarity (reviewed in [13,14]).

It is worth mentioning that the biogenesis pathway is under a strict, very comprehensive regulation [15] and that one of the factors that affects the miRNA biogenesis machinery are the miRNA molecules themselves [16]. miR168 is present in all plants and acts as a regulator of *AGO1* [17]; while, in Arabidopsis and *Physcomitrella patens*, miR162 negatively regulates the *DCL1* [18]. Interestingly, in ancient plants, the regulation of *DCL1* by miR162 was not present and the miR162 target site in the *DCL1* gene sequence seems to have been gained during plant evolution [19]. 

A few years ago, ncRNA was sometimes called a junk RNA, but now we know that ncRNA plays a tremendous role in plant development in all/almost all plant processes [20]. miRNAs play crucial roles in most aspects of plant growth and development including the response to biotic and abiotic stresses and regulating embryogenesis (reviewed in [21,22]). Embryogenesis is a key developmental process that leads to the generation of a morphologically simple plant that is composed of only the most basic features such as the precursors for all the major tissues and stem cells. Embryo formation can be initiated from either a zygote after the fusion of the gametes (zygotic embryogenesis, ZE) or from the asexual embryos that are generated from somatic cells (somatic embryogenesis, SE), which is usually induced in vitro [23,24]. Zygotic embryo development in the dicot model plant Arabidopsis is well described and is a perfect model system to investigate the function of miRNAs in embryogenesis. Arabidopsis embryos undergo a simple and predictable pattern of stereotypical cell divisions during ZE and their development can be divided into eight different phases: preglobular, globular, early heart, late heart, early torpedo, late torpedo, bent cotyledon, and mature green [25,26,27]. Each of the ZE stages has morphogenic specific features and a stage-specific modulation of distinct miRNAs sets with a particular pattern expression that is responsible for its precise functions during the different phases of ZE in dicots and monocots [28,29,30], so the validation of miRNAs role in the ZE process should be examined specifically in every distinct stage.

Even though somatic embryos during development seem to progress through similar morphogenic stages as their zygotic counterparts including the globular, heart-shaped, torpedo, and cotyledonary stage [25,31,32], somatic embryos lack the endosperm and seed coat tissues [33], which are essential for ZE, while the conditions during in vitro cultures on hormone-rich media may cause the misidentification of the miRNAs that are specific for somatic embryos from those that are regulated in response to the application of an exogenous growth factor. The evidence on the stage-specific modulation of miRNAs during SE has also been observed in Arabidopsis, cotton, and coconut [34,35,36]. Notwithstanding, the analyses that are performed are usually limited to the embryogenic vs. non embryogenic tissue or to the induction vs. developmental stage of SE. Lastly, a very promising paper which refers to the suspensor-derived SE system in Arabidopsis has been published [37], which could help overcome the problem with tissue heterogeneity taking to SE analyses. In the mentioned system, the cells undergoing SE are easy to identify so the performed analyses could be done on very specific, homogeneous tissue fraction of somatic embryos. To date, miRNA analyses have not been performed on selectively isolated somatic embryos in different stages of development without non-embryogenic explant tissue. Nevertheless, comparisons of function of miRNAs in somatic versus zygotic embryos in the future may reveal an miRNA-based regulation network of the embryonic differentiation events, with common or specific miRNAs to both processes. In the ongoing debate about the ZE and SE similarities, recent analyses of an embryogenic culture of Arabidopsis and *Pinus pinaster* showed that the SE transcriptome seems to be distinctly different from the transcriptome of a zygotic embryo [38,39]. That finding highlighted how important the precise identification of the function of miRNAs in each of the different phases of both analysed processes is, and the miRNA-dedicated tools for miRNA functional analysis can make it possible.

**Figure 2:**
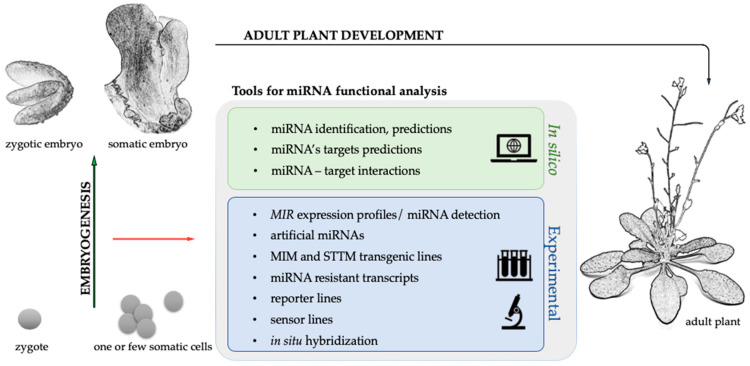
The toolbox dedicated for the functional analysis of plant miRNAs during embryogenesis using the in silico and experimental approaches.

In the face of the totipotency that is responsible for what governs a cell to become an embryo, what limits the switch of the cells to embryogenesis, no matter what kind, is an important question. Even in in vitro induced SE, not all cells can be reprogrammed. In fact, only a small number of cells undergo a complete reprogramming. A major issue in plant developmental analysis is to unravel the mechanisms that operate during embryogenesis that enable a plant to specify its body plan through tissue differentiation patterns. Research in the last decade has demonstrated that miRNAs have crucial roles during plant embryogenesis [40]. Plant miRNAs tend to target the genes encoding the key developmental regulators among which are many transcription factors (TFs) [41], which have been described as extremely important for ZE and SE [41,42]. Accordingly, Arabidopsis zygotic embryos, which lack the DCL1 enzyme, arrest early in development [41] also, other miRNA biogenesis enzymes and, by implication, miRNAs, seem to be important for the embryonic cell differentiation from the earliest ZE stages including suspensor and embryo development [43,44,45,46]. From the other side, it has been noticed that somatic explants of a *dcl1* mutant are unable to initiate embryogenic induction in vitro [47] suggesting a highly important role of miRNAs also during SE induction and somatic embryos development. Therefore, a significant part of the knowledge of miRNA engagement in plant development is based on an analysis of transgenic lines that have a modulated expression of the miRNA biogenesis-related genes [3]. However, the functions of individual miRNA–target interactions remain largely unknown. A functional analysis of miRNA molecules and their targets is challenging due to the existence of multigene families of *MIRs* that often have redundant functions. The functional diversity of the genes that are targeted by one mature miRNA adds further complications. Moreover, difficulties in the interpretation of the results that are obtained from the expression profiling of *MIRs*, are related with the diverse modes of the miRNA regulation of a target gene, which involves the cleavage of the target mRNA or the inhibition of mRNA translation. In the concept of ZE or SE functional analysis, a few additional aspects should be considered. Zygotic embryos are deeply embedded within the maternal tissues, and process through the developmental stages with very specific features also in terms of miRNAs activity. Moreover, the early stages of somatic embryo development are very tricky to analyse due to the difficulty in distinguishing the cells that are triggered towards embryogenic development from the other explant cells. Although, some of the problems can be overcome by using the miRNA-dedicated tools, (i) databases for in silico analysis; (ii) miRNAs detection and expression approaches; (iii) reporter and sensor lines for a spatio-temporal analysis of the miRNA-target interactions; (iv) in situ hybridisation protocols; (v) artificial miRNAs; (vi) *MIM* and *STTM* lines to inhibit miRNA activity; and (vii) the target genes resistant to miRNA (Figure 2), which are described in the next paragraph, and most of them have been optimised for analyses of embryogenesis.

## 2. Available Plant miRNA-Dedicated Research Tools

### 2.1. In Silico Analysis

Often the very first steps in the miRNA analysis are taken in front of a computer screen; the popularity of such solutions is proven by the number of citations of publicly available databases sources, which can be counted in the thousands. There is a wide variety of databases and online tools for miRNA in silico analysis (Table 1), which has been collected in the tools4miRs platform [48] (reviewed in [49]) and led, among others, to predictions of novel miRNAs, miRNA targets, or miRNA-target interactions. Some of the available databases are general such as the largest miRNA database: miRbase [8], whereas others are specific, for example, only collecting the interactions between miRNAs and TFs: TransmiR [50] or are dedicated for medicinal plant miRNAs: MepmiRDB [51] and grapevine miRNAs: miRVIT [52] (Table 1). More than ten years ago when the bioinformatics that is known today was evolving, computational analysis was used to identify novel and conserved miRNA molecules [53,54]. Today, bioinformatic analyses are key components of high-throughput NGS (next generation sequencing) analysis such as RNA-seq or small RNA-seq (sRNA-seq) and without them the genomic approaches would be unthinkable. Advancements in the molecular and computational approaches has not only resulted in exponential growth in the discovery and study of sRNA but has also provided a deeper insight into the miRNA regulatory networks. With the accumulation of huge sRNA sequencing datasets from sRNA-seqs, it is almost impossible to analyse every sequence experimentally; however using the bioinformatics tools and databases enables huge data sets to be analysed in a short time with minimum costs and without compromising on the specificity of the analysis [55,56,57].

### 2.2. miRNA Profiling—Isolation, Detection, and Quantification Methods

By taking advantage of in silico analysis, identifying a miRNA, miRNA target, or miRNA-target pair that is engaged in the process of interest could be simple, rapid, and productive. However, a scientific hypothesis that is based on in silico analysis should be validated.

Once a golden standard, the oldest method for miRNA detection is the Northern blot [5], which can not only be used for discovery but also for the validation and expression of miRNAs. To date, a variety of Northern blot protocols have been established with RNA or DNA radio-labelled and fluorescent oligonucleotides as well as with LNA probes (lock nucleic acid), which have a high hybridisation affinity [71,72].

Nowadays, there is a tendency to use the RT-qPCR (quantitative reverse transcription PCR) methods for detecting miRNAs and analysing their expression, especially to verify other methods such as the Northern blot. The detection of miRNAs is affected by their small size and the lack of a poly(A) tail and 3′ end-modifications. To avoid these limitations, the stem-loop RT-qPCR method has been designed to detect and quantify mature miRNAs in a precise and reliable manner. This method is based on a miRNA-specific stem-loop RT primer that is hybridised to miRNA and then reverse transcribed. The RT product can then be amplified and monitored in a qPCR reaction using a miRNA-specific forward primer and a universal reverse primer. The developed protocols enable the profiling of mature miRNA accumulation and high-throughput analysis of the miRNA expression, which has been successfully used in the functional analysis of miRNA during ZE and SE [35,73,74,75,76,77].

The availability of many plant-dedicated protocols [73,78,79,80] and commercial kits (miScript Plant RT Kit, TaqMan Assay, and the *mirVana^TM^* qRT-PCR-miRNA Detection Kit) offers a picture of the popularity of the RT-qPCR technique. It is impossible to find a study that describes the miRNA-target role in plant embryogenesis without a single RT-qPCR analysis, which is also broadly used as a control for the results obtained from high-throughput data such as microarrays and NGS. miRNA profiling using NGS has revolutionised miRNA analysis and there are still new instruments, sequencing platforms, and new methods that appear, such as sRNA-seq using single-cell sequencing [81] which provide new opportunities for analyses also during embryogenesis. Until now, sRNA-seq analyses have been performed during embryogenesis in *Picea sprus* [82], *P. pinaster* [39], *Picea glauca* [30], *Triticum aestivum* [83], Arabidopsis [28,30,35,38,41,84], *Dimocarpus longan* [85], *Zea mays* [86], *Citrus sinensis* [87], *Larix leptolepis* [88], *Gossypium hirsutum* [89,90], *Phyllostachys heterocycla* [91], *Oryza sativa* [92], and *Lilium pumilum* [93] (for SE reviewed in [94]). Plant embryogenesis is challenging to analyse due to the small, early embryos that are deeply embedded in the maternal tissues, which often results in RNA contamination from maternal tissue, and due to difficult distinguishing of the cells that are undergoing SE within an explant tissue, protocols, which are specifically dedicated for ZE and SE, have been developed and successfully used for miRNA analysis [28,38,84,95]. Since the low-input small RNA sequencing (sRNA-seq) method, which can be used to generate the profiles of miRNAs from as little as one to five ng of RNA and INTACT (isolation of nuclei tagged in specific cell types) and FANS (fluorescence-activated nuclei sorting) which permit the isolation of nuclei from cells that are undergoing SE or ZE from the majority of non-embryogenic cells from an explant, it makes a high-throughput analysis during embryogenesis much more accurate [28,84,96,97,98,99]. It is worth mentioning the requirement to share the data from NGS during the publication process, which ensures access to big data without performing an experiment. Lastly, an online website, Arabidopsis Small RNA Database (ASRD, http://ipf.sustech.edu.cn/pub/asrd), which permits more than 2000 publicly available Arabidopsis sRNA libraries to be queried, has been developed [100].

In addition to selecting the appropriate miRNA detection methodology, another bottleneck is the RNA isolation step, which is highly relevant and is both species- and tissue-dependent. RNA or dedicated kits/methods for isolating the sRNA fraction are available to analyse the miRNA expression in explants/tissues that are undergoing SE or ZE. The *TRIzol*™ Reagent is primarily used in studies of *D. longan* [85,101], *Hordeum vulgare* [102], and Arabidopsis [62,63,79]; Quick-RNA MiniPrep is used in studies of *Z. mays* [86,103]; Plant/Fungi Total RNA purification is used in studies of *P. pinaster* [39]; C-TAB is used in studies of *L. pumilum* [93], and the *mirVana^TM^* miRNA Isolation Kit is used in studies of Arabidopsis [35,84], *Acacia crassicorpe* [104], and *T. aestivum* [83] to isolate RNA with an sRNA fraction to analyse miRNAs during embryogenesis.

### 2.3. Monitoring the MIR/miRNA Localisation and Activity

**Figure 3:**
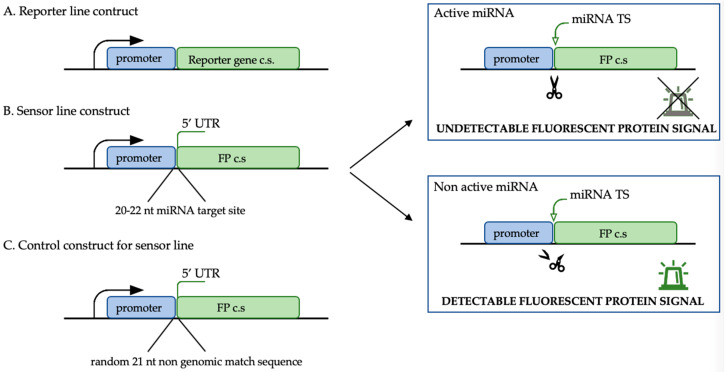
The constructs that were used for the spatio-temporal analysis of the miRNAs; (**A**) the reporter line construct with the promoter of choice (usually a promoter of the *MIRNA* gene); (**B**) the miRNA sensor line construct that was used for the analysis of miRNA activity with the miRNA target site and the control construct (**C**) for the sensor line analysis. FP: Fluorescent protein; c.s.: Coding sequence.

*MIR* expression can be spatio-temporal depending on the species, organ, tissue, developmental stage, or stress conditions, which has been described, among others, in Arabidopsis, tobacco, soybean, rice, and wheat [105,106,107,108,109,110]. The precise localisation of mature miRNAs in a tissue or organ is extremely important for understanding the biological function of the miRNA molecules. The fact that the SE induction occurs in the upper part of explants, preferentially on the adaxial side of the cotyledons; somatic embryos can be of a single-cell or multicellular origin and they develop asynchronously from both the protodermal and subprotodermal cell layers [31,111]; and heterogeneous cell populations of explants undergoing ZE and SE must all be taken into account, which often makes the results that are obtained from transcriptomic analysis on whole explants difficult to interpret. Therefore, identifying the embryogenesis-associated miRNAs or genes requires insight into the spatio-temporal expression patterns of the candidates. Usually, a determination of the spatio-temporal expression patterns of miRNAs relies on indirect detection using the reporter lines that have *MIR* promoter fusions to GUS (glucuronidase) or fluorescent proteins such as GFP or YFP (Figure 3A). *MIR* reporter lines have been used in the functional analysis of miRNAs: miR160, miR166, miR167, miR319, miR393, and miR396, which are engaged in the regulation of embryogenic potential in the somatic cells and zygotic embryo development in Arabidopsis [28,112,113,114,115,116,117]. However, small RNAs such as miRNA can move over short and long distances within cells, tissues and organs and localizing it may be different than finding the site of the corresponding *MIR* gene expression [118,119,120,121]. The overwhelming majority of reporter lines monitor a promoter activity rather than lead to the localisation of the corresponding transcript, in this case, mature miRNA. Moreover, transgenic reporter-based methods are limited due to the time-consuming steps of transgenic plant generation. Methods such as sRNA in situ on sections or whole mount in situ (WISH), which are designed for sRNA, including miRNA, that has been optimised and used for the functional analysis of miRNA in developing zygotic and somatic embryos seem to be more accurate for the functional analysis of miRNAs. The developed protocols are based on digoxigenin or fluorescent-labelled LNA probes and an sRNA-specific post-fixation step using EDC as a cross-linker to increase the sensitivity and specificity of the hybridisation. It is even possible to co-localise two sRNA molecules in one sample, which has been described during the development of anthers in maize and litchi. The probe can be a sequence of a mature miRNA molecule or mRNA that is targeted by the candidate miRNA [115,122,123,124,125]. Those methods enabled the localization of the miR156, miR167, and miR390 accumulation and the engagement of miR160 and miR165/166 in the acquisition of embryogenic capacity in Arabidopsis somatic cells to be determined [115,126] and miR160, miR166, miR167, miR172, and miR390 in the bent cotyledon zygotic embryos in Arabidopsis to be detected [122]. The WISH-investigated accumulation of mature miR390 has been also linked with lateral root and primary root meristem formation that has a genetic convergence into in vitro tissue dedifferentiation and callus formation [127], which covers the hypothesis of miR390 engagement in the developing somatic embryos that is based on the results of the WISH [115].

The discovery of the site of miRNA accumulation was a big step forward in acquiring knowledge about the biological function of the analysed miRNA, while the sensor lines have been developed to establish the miRNA activity in the tissue/cells in which it is accumulated. The design of a sensor construct contains a 20-22-nt miRNA target site is in the 5′UTR region of a GFP coding sequence under the promoter of choice (Figure 3B) [41]. An almost identical construct without the miRNA target site (the gap is filled by 21-nt non-genome matching sequence) is used as the control (Figure 3C). The expected observation is that a weaker GFP signal will be produced by a transgenic sensor line in the tissue in which the promoter is expressed and that miRNA will mediate the target repression than in the control in which the GFP signal is not disrupted by the miRNA activity. To date, transgenic sensor lines have been used to validate the miR156 repression of *SPL10* and *SPL11* (*SQUAMOSA-PROMOTER BINDING PROTEIN-LIKE*), miR160-*ARF17* (*AUXIN RESPONSE FACTOR17*), miR165/166-*PHB* (*PHABULOSA*), miR167-*ARF8,* and miR319-*TCP4* during the development of the Arabidopsis zygotic embryo and ovule [28,41,114]. In addition to their use in the miRNA functional analysis in embryogenesis, sensor lines have also been successfully used to verify the role of miR156-*SPL10* in the root meristematic cells and in root-derived shoot regeneration in Arabidopsis [128], which are processes that genetically resemble in vitro induced embryogenesis from somatic cells [127]. Moreover, sensor constructs have been used in miR156-*SPL* and miR159-*MYB* analyses in *Rosa hybrida* petals [129]. Sensor lines are broadly used in functional analyses of miRNA molecules in mammalian cells, for example, to simultaneously analyse the high-throughput miRNA activity for hundreds of miRNAs using a Sensor-seq assay [130]. This type of reporter sensor lines might be very helpful for the miRNA functional analysis because it reveals the time and site of the miRNA target repression.

### 2.4. Artificial miRNA Molecules

**Figure 4:**
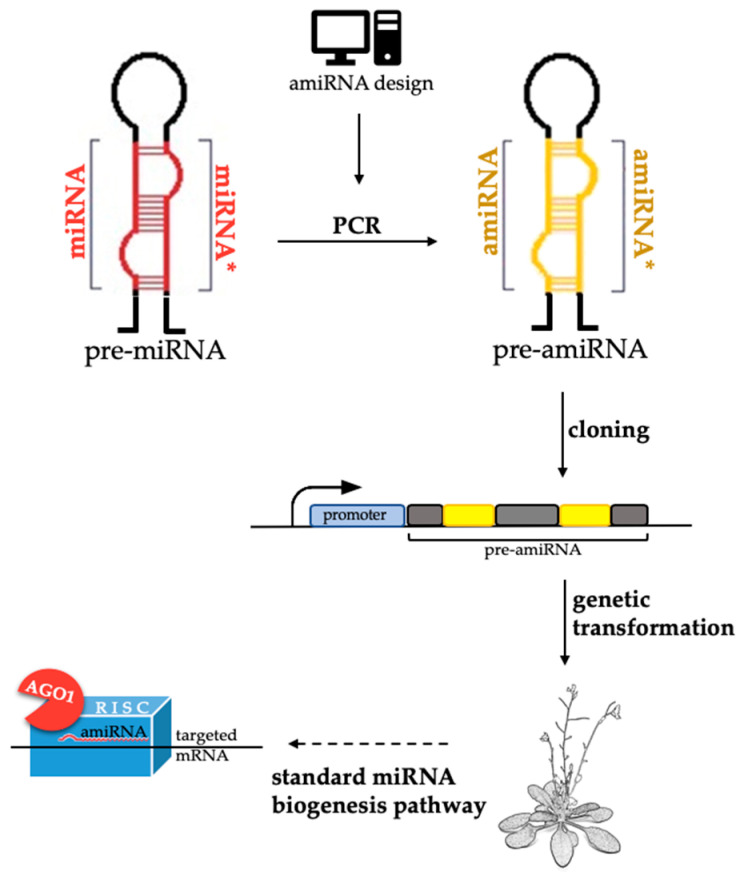
A schematic representation of the artificial miRNA (amiRNA) design, introduction into the plant genome and mode of action. pre-miRNA: Precursor-miRNA; pre-amiRNA: Precursor-amiRNA; AGO: ARGONAUTE; RISC: RNA Induced Complex.

Artificial miRNAs (amiRNA) are extensively used in the field of plant molecular biology as a versatile tool of RNAi methods. Their mode of action is based on the miRNA repression of a targeted gene expression, which is considered to be one of the highly conserved mechanisms in the plant kingdom (reviewed in [131]). Similar to miRNAs, amiRNAs are short 21 nt, single-stranded sRNA molecules that are generated from endogenous pre-miRNA structures (Figure 4). The mature miRNA sequences of the miRNA/miRNA* duplex within the pre-miRNA have to be replaced by the designed amiRNA/amiRNA* sequences. The construct that is generated under the promoter of choice that is cloned into a vector has to be introduced to a plant via a genetic transformation procedure. Then, the built-in amiRNA sequence in the endogenous pre-miRNA structure is processed through the standard miRNA biogenesis pathway. The first protocols for amiRNA design were published only four years after the discovery of miRNAs in plants [132] and since then, new solutions that increase efficiency, cut the cost, time consumption, and broaden the spectrum of applications of this technique are constantly appearing [133,134,135,136,137].

The design of a candidate amiRNA that is to be used for analysis has been simplified and automated by a variety of web tools for amiRNA design such as Web MicroRNA Designer (WMD3, http://wmd3.weigelworld.org/cgi-bin/webapp.cgi), AmiRNA Designer (http://www.cs.put.poznan.pl/arybarczyk/AmiRNA/), and the Plant Small RNA Maker Suite (P-SAMS, http://p-sams.carringtonlab.org) and there are published protocols that provide a step-by-step procedure and the necessary oligonucleotide sequences for a broad range of mono- and dicot-plant species [132,138,139,140,141]. amiRNAs are commonly used as an alternative to the knockout transgenic lines. Since the redundant multi-gene families of *MIRs* which additionally are short, there are difficulties in generating *MIR* knockout mutants, except for a few miRNAs, the advantages of amiRNAs has been applied to repress endogenous *MIR* genes [142]. The application of the amiRNA approach enabled the repression of one or an entire family of *MIRs*. At this time, only two analyses have been performed on zygotic or somatic embryos using the amiRNA approach. One of them presented amiRNAs as a valuable tool in the functional analysis of miR164, miR165/166, miR167, and their targets in Arabidopsis, tomato, and tobacco during the embryonic meristem establishment in zygotic embryos, the differentiation of the lateral organs, vascular development, flowering, and cell growth [143], while the other showed the functionality of amiRNAs that were designed based on the Arabidopsis pre-miR319 in targeting the coat protein of *Grapevine fanleaf virus* (GFLV) in grapevine somatic embryos. GFLV causes a fanleaf degeneration disease, which is a major threat to grapevine production and amiRNA seems to be useful for engineering GFLV-resistant grapes in the future [144]. Lastly, the company Thermo Fisher Scientific introduced the *mirVana*™ miRNA inhibitors, which are chemically modified, single-stranded RNA molecules that are designed to specifically bind to and inhibit endogenous miRNA and to enable the miRNA functional analysis by downregulating the miRNA activity. These ready-to-use miRNA inhibitors, in other words, the amiRNAs are designed for a wide range of plant species and miRNA families. The search tool for a candidate sequence is easy to use and is available online at the company website (https://corporate.thermofisher.com/en/home.html). Furthermore, the application of amiRNA could become a promising tool for determining the contribution of specific miRNA in embryogenesis and other biological aspects of plant development, although the generation of transgenic plants is a significant limitation.

### 2.5. Target Mimicry—A Method to Regulate the miRNA Activity

**Figure 5:**
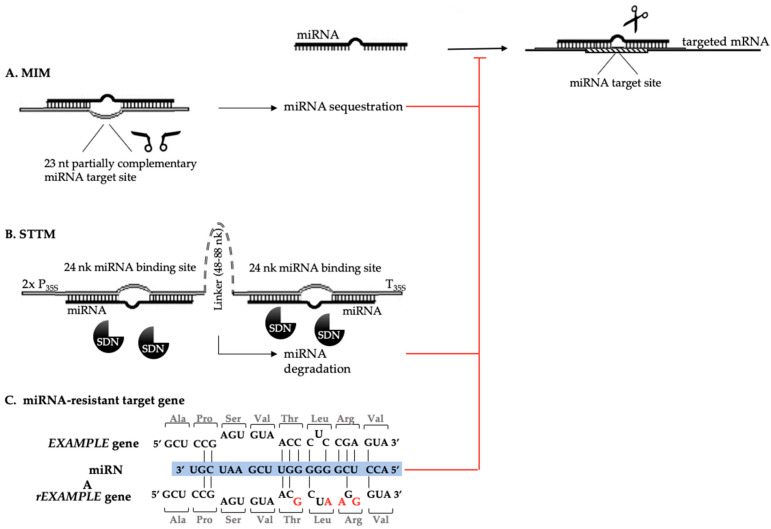
The methods that were used to alter the miRNA activity in the plants; (**A**) the MIM and (**B**) STTM constructs that were based on target mimicry, which led to the sequestration or degradation of the miRNAs by the SDNs. (**C**) The synonymous mutations (in red) that were introduced into the *EXAMPLE* gene coding sequence, which resulted in a miRNA-resistance of the modified *mEXAMPLE*. MIM: Mimic; STTM: Short tandem target mimic; P: Promoter; T: Terminator; SDN: Small RNA degrading nucleases; m: Modified.

A promising alternative technology for the *MIR* knockdown lines was developed in 2007 when Franco-Zorrilla et al. described the miRNA target mimicry (TM), an endogenous mechanism for the transcriptional regulation of miR399 activity in Arabidopsis [145], which are also called miRNA decoys, sponges, or competing endogenous RNAs (ceRNAs), and were later also found for other miRNAs in Arabidopsis and rice [146]. The mechanism is based on the presence of the long non-protein coding mRNA (lncRNA) that carries the 23-nt partially complementary target site for miRNA, which led to the sequestration of this miRNA and the arrest of its activity for the correct miRNA target, after which the decrease in miRNA simultaneously led to an increased expression of the miRNA targets (Figure 5A). Today, a TM database PeTMbase (http://petmbase.org) that collects the endogenous TMs for 11 plant species [147], which makes the miRNA functional analysis easier, is available. A few years after the discovery of miRNA TM [145], Yan et al. described a technique based on TM but with an even higher efficiency in silencing the miRNA activity—the short tandem target mimic (STTM) [148,149]. The concept of STTM is based on the specific structure of the construct that is built with two short, 24-nt non-cleavable miRNA target sites that are separated by a 48-88-nt linker, which leads to the degradation of the targeted miRNAs by small RNA-degrading nucleases (SDNs) (Figure 5B).

The first contrivance of TM activity was used to generate a large-scale collection of *MIM* lines for 73 *MIR* families in Arabidopsis. Using the target-mimicry approach to reduce miRNA for a loss-of-function analysis led to the successful knockdown activity of many miRNAs [150]. It is worth mentioning that the miRNA target site is not the only key element in the TM sequence. A recent study showed that minor mutations (nucleotide substitutions) in the flanking sequences of the miRNA binding sites in lncRNA that have serving as a backbone for TM can strongly enhance or reduce (target miRNA-dependent) TM-miRNA interaction and thus the effectiveness of the method [151]. An analysis of functions of miRNAs using the *MIM* lines showed that miR167, which regulates *ARF6* and *ARF8* may be engaged in ZE as *MIM167* seeds were retained in the siliques and had an impaired development [150]. The hypothesis about the involvement of miR167 in regulating zygotic embryo development was also indicated in other analyses in Arabidopsis [28,41,152,153] and loblolly pine [29]. The importance of miR167-ARF6/ARF8 in the acquisition of embryogenic competence during SE has also been indicated in cotton by a decrease in the miR167 activity, which led to enhanced callogenesis and an increased production of somatic embryos [154], while an overexpression of *MIR167* genes inhibited the formation of somatic embryos in Arabidopsis, which showed that miR167 negatively regulates SE induction [112]. Moreover, Lin et al. identified the expression of two endogenous TM transcripts that modulate the miR167 activity during SE in *D. longan*, which resulted in increased expression of *ARF6* and *ARF8*, thus indicating the important role of miR167-ARF6/ARF8 in the development of somatic embryos [155], which is in line with previous analyses that were carried out using other available functional genomics tools ([75,156,157] reviewed in [94]). Moreover, the TM approach has been successfully used for the functional analysis of miR156, miR160, miR166, miR393, miR396, miR398, and miR1432, thereby indicating their importance in regulating sexual and asexual embryogenesis in barley [102], tomato [158,159], rice [160,161], and Arabidopsis [115,126,162,163]. Note that MIMs and STTMs have the advantage of being able to target miRNAs from multiple redundant *MIR* genes, but that they also have a disadvantage that may not result in a complete loss of function, especially in MIMs.

### 2.6. Transcripts That Are Resistant to miRNA Cleavage

The role of miRNA is usually indirectly elucidated based on knowledge about the function of their target genes. The miRNA-target transcripts that are resistant to the miRNA action can be used to unravel the relationship between miRNA and putative targets. Multiple silent mutations are created within the miRNA binding site of a candidate target gene, which gives it the resistance to miRNA regulation (Figure 5C) [164]. Then, a functional analysis of miRNA-mediated regulation is elucidated by comparing the expression effect of a miRNA-resistant target vs. its wild-type counterpart. It seems that the first miRNA-resistant line was generated accidentally via EMS mutagenesis before the discovery of plant miRNA. The *phb* gain-of-function mutant phenotype is believed to be caused by *PHB* mRNA resistance to the miR166-directed cleavage, which results in the overexpression of *PHB* [165,166,167]. Unintentionally, that mutant also became the first use of miRNA-resistant target mRNA in a miRNA functional analysis of the development of zygotic embryos in Arabidopsis [166,167] and recently during SE induction [126]. Later, this approach was applied to describe the engagement of miR156/157, miR160, miR164, miR165/166, miR167, miR319, miR393, and miR396 in ZE regulation in Arabidopsis and rice [28,41,113,117,168,169,170,171,172,173]. Except for the *phb1* line, there are only a few examples of using miRNA resistance in the SE analysis. An investigation of the ability of the *mARF16* line, which carries the miR160-resistant form of *ARF16*, to acquire an embryogenic potential contributed to the description of an important role of miR160 in regulating the developmental plasticity of Arabidopsis cells under in vitro conditions [126]. Additionally, an analysis of a transgenic line that had an overexpression of a miR393-resistant form of *TIR1* (*TRANSPORT INHIBITOR1*, *mTIR1*) showed an enhanced auxin sensitivity and pleiotropic effects on plant development including the overproduction of lateral roots [174], which was caused by the suppression of the miR393 activity. The significance of the auxin sensitivity that is regulated by miR393 and *TIR1* has also been indicated during SE induction in Arabidopsis. Moreover, the indirect use of the miRNA-resistant line in a functional analysis of SE may be elucidated based on a functional analysis of miR847, which targets the *IAA28* encoding auxin-responsive protein. The miR847 cleavage-resistant mutant *mIAA28* showed the engagement of the miR847-*IAA28* pair in regulating meristematic competence, which determines the duration of cell proliferation and lateral organ growth in Arabidopsis [175]. Moreover, the target genes mutated at their miRNA target site and thus became less sensitive to miRNA inhibition, have been linked with reporter genes and represent a powerful approach for unravelling the contribution of miRNAs and their targets during the ZE and SE processes in a spatio-temporal manner. The *pPHB::muPHB-GFP* line, which carries the mutated, version of the *PHB* transcript resistant to the miR165/166 cleavage, was used to investigate the PHB signal with and without the miR165/166 regulation in the developing zygotic embryos, ovule, and in the somatic cells undergoing the embryogenic transition that had been induced in vitro in Arabidopsis [113,114].

The fact that miRNAs usually have more than one target and that the overexpression of a miRNA-resistant target may not reveal a complete picture of the miRNA functions and phenotypes corresponding to transgenic artifacts must be considered [176].

## 3. Conclusions and Future Perspectives

Although credible functional analyses of the role of the miRNA molecules in regulating ZE and SE may be tricky, nowadays with the variety of specific miRNA-dedicated tools, they are within reach. All the methods mentioned in this review have some advantages and limitations, making a decision of choosing a proper research tool the crucial step on the way to obtain the credible and reliable results. There is no gold standard in the preparation of a miRNA’s functional analysis ‘pipline’ for ZE or SE processes. Based on the analysed species, specific miRNAs or/and targeted genes, and stage of the ZE/SE process, the proper tools should be considered. In my opinion, the best toolbox to use in order to find and check the engagement of miRNAs in ZE or SE in a model plant would be isolation of the sRNA fraction from as much specific tissue fraction as possible, for example, from the manually isolated embryos [97,177] or from a cell type-specific fraction obtained by fluorescence-activated cells or nuclei sorting (FACS; FANS) or INTACT approaches [26,84,96]. Then, the low-input sRNA-seq can be performed [28] and the obtained results should be randomly validated by the real-time quantification preceded by stem-loop RT-PCR [76,77]. In the next steps, the localization of the candidate miRNA should be examined in different developmental stages of ZE/SE by in situ hybridisation with LNA probes and EDC cross-linking [115,122,125], and their activity by using the miRNA sensor lines should be verified [28,41]. To reveal the function of the candidate miRNA the examination of *MIR* overexpression lines or amiRNA lines [132] to validate the effect of accumulation of miRNA vs. STTM lines [161] (for whole miRNA families) or the lines harboring miRNA-resistant targets [164] resulting in the abolition of the miRNA function should be performed.

Generally, analyses of the miRNAs should be comprehensive and a multitool that combines, e.g., miRNA spatial and temporal analysis, miR-resistant target constructs, and miRNA mimicry, to unravel the miRNA-target module in the investigative process. To date, the variety of functional genomic miRNA’s research tools have been used to unravel the engagement of miRNAs in zygotic and somatic embryogenesis process and within them the approaches that are based on in situ hybridisation and mimicry have been used most often. All the research tools/methods discussed in this review, that have been used in the ZE and SE analysis of miRNAs and their target genes are summarized in Table 2. However, despite major efforts of scientists over the past several years, a complete miRNA functional characterisation remains an inscrutable map that is yet to be explored. In the future, the CRISPR-Cas9 (clustered regularly interspaced short palindromic repeats, CRISPR-associated protein9 (Cas9)) gene-editing technology will probably be feasible for systematically generating *MIR* knockout mutants to study their roles in regulation of plant embryogenesis. To date, CRISPR has been used to knock out the *MIR1514, MIR1509, MIR815, MIR820, MIR169,* and *MIR827* genes in soybean, rice, and Arabidopsis, respectively [178,179,180].

It is worth mentioning the usefulness of the described tools not only in miRNA functional analyses but also as an effective approach for improving the agronomic traits in model and crop plants (reviewed in [181,182,183]).

## Figures and Tables

**Table 1:** The publicly available plant miRNA databases and web tools for in silico analyses [8,48,50,51,52,58,59,60,61,62,63,64,65,66,67,68,69,70]. WT: Wild type; R: References; E: Expression; I: Interaction.

Name	miRNA	Target	I	E	Updated	Website	Additional Information	R
tools4miRs	✓				III 2020	https://tools4mirs.org	Platform gathering methods for miRNA analysis	[28]
miRBase	✓				X 2018	http://www.mirbase.org/	The biggest miRNA database	[11]
MicroPC	✓	✓			X 2018	http://www3a.biotec.or.th/micropc/	Predicting and comparing plant miRNA	[36]
PmiRKB	✓	✓	✓		X 2018	http://bis.zju.edu.cn/pmirkb/	Plant miRNA knowledge database	[37]
PNRD	✓	✓	✓		X 2018	http://structuralbiology.cau.edu.cn/PNRD/index.php	Comprehensive analysis platform for plant ncRNAs	[38]
miRTarBase	✓	✓	✓		X 2018	http://mirtarbase.cuhk.edu.cn/php/index.php	miRNA-target interactions for Arabidopsis and rice	[39]
MepmiRDB	✓	✓			X 2018	http://mepmirdb.cn/mepmirdb/index.html	A medicinal plant microRNA database	[30]
miRVIT	✓	✓			X 2018	http://mirvit.ipsp.cnr.it	Novel grapevine miRNA	[31]
TransmiR v2.0	✓	✓			X 2018	http://www.cuilab.cn/transmir	Transcription factor – miRNA regulations	[29]
PASmiR	✓	✓			X 2018	http://pcsb.ahau.edu.cn:8080/PASmiR	miRNA regulation in abiotic stress	[40]
DPMIND	✓	✓	✓		X 2018	http://cbi.njau.edu.cn/DPMIND/	Degradome-based miRNA-Target interaction	[41]
psRNAtarget	✓	✓			X 2018	http://plantgrn.noble.org/psRNATarget/	sRNA target analysis	[42]
WPMIAS			✓		X 2018	https://cbi.njau.edu.cn/WPMIAS/	Validation of predicted interactions of miRNAs-target	[43]
TAPIR		✓			X 2018	http://bioinformatics.psb.ugent.be/webtools/tapir/	Target prediction for plant miRNA	[44]
ENCORI	✓		✓		X 2018	http://starbase.sysu.edu.cn	sRNA interactions	[45]
PmiRExAt	✓			✓	X 2018	http://pmirexat.nabi.res.in/index.html	miRNA expression in wheat, maize, rice, Arabidopsis	[46]
miRNEST2	✓	✓	✓	✓	X 2018	http://rhesus.amu.edu.pl/mirnest/copy/home.php	Integrative resource of miRNA-associated data	[47]
miREX2	✓		✓	✓	X 2018	http://www.combio.pl/mirex	Expression of miRNAs from WT and mutants	[48]

**Table 2:** The tools that have been successfully used in the functional analyses of miRNAs during zygotic (ZE) and somatic embryogenesis (SE). Some of the analyses are considered to be indirect (~) approaches for revealing the function of miRNAs in the mentioned process. amiRNA: Artificial miRNA; STTM: Short tandem target mimic; MIM: Mimic; eTM: Endogenous target mimic; rGEN: miRNA-resistant target gene.

Investigated miRNA Feature	Method	miRNA	miRNA-Target	Species	ZE/SE	Reference
LOCALIZATION	*MIRNA* Reporter	miR165/166, miR167	-	*A. thaliana*	ZE	[112,113,114,117,153]
		miR167, miR393, miR396	-	*A. thaliana*	SE	[47,115,116]
	in situ Hybridisation	miR156, miR160, miR166, miR167, miR390	-	*A. thaliana*	SE	[115,126]
		miR156/157, miR158, miR159, miR160, miR161, miR166, miR167, miR168, miR169, miR172, miR390, miR396, miR449, miR472	-	*A. thaliana*	ZE	[28,122,153]
LOCALIZATION & ACTIVITY	miRNA Sensor	miR156/157, miR160, miR165/166, miR167, miR319	*SPL10,SPL11; ARF17;PHB; ARF8;TCP4*	*A. thaliana*	ZE	[28,41,114]
		miR156, miR159	*SPL7;MYB (RU13577)*	*Rosa rugosa*	~ZE	[129]
		miR156	*SPL10*	*A. thaliana*	~SE	[128]
	mGENE+GUS	miR167	*ARF6, ARF8*	*A. thaliana*	ZE	[117]
	mGENE+GFP	miR165/166	*PHB*	*A. thaliana*	ZE	[113,114]
		miR165/166	*PHB*	*A. thaliana*	SE	[126]
ACTIVITY	amiRNA	miR164, miR166/166, miR167	-	*A. thaliana*	ZE	[143]
		miR164, miR165/166	-	*Solanum lycopersicum*	ZE	[143]
		miR319	-	*Vitis Vinifera*	SE	[144]
	MIM	miR156, miR157, mir159, miR160, miR164, miR165/166, miR169, miR170, miR171, miR172, miR319, miR393, miR394	-	*A. thaliana*	ZE	[150]
		miR393	-	*Hordeum vulgare*	ZE	[102]
		miR167	-	*Gossypium* *arboreum*	ZE	[154]
	eTM	miR167	-	*Dimocarpus longan*	SE	[155]
	STTM	miR160, miR396	-	*S. lycopersicum*	ZE	[158,159]
		miR160, miR172, miR398, miR1432	-	*Oryza sativa*	ZE	[160,161]
		miR165/166	-	*A. thaliana*	ZE	[162,163]
		miR156, 165/166	-	*A. thaliana*	SE	[115,126]
	mGENE	miR156, miR160, miR164, miR166, miR167, miR319	*SPL10, SPL11; ARF10, ARF17; CUC1; CUC2; PHB; ARF6, ARF8; TCP4*	*A. thaliana*	ZE	[28,41,117,152,166,167,168,170,171,173]
		miR160, miR396c	*ARF18; GRF4*	*O. sativa*	ZE	[169,172]
		miR160	*ARF10*	*A. thaliana*	SE	[126]
		mir847	*IAA28*	*A. thaliana*	~SE	[175]

## Abbreviations

AFB1	AUXIN F-BOX PROTEIN1
AGO	ARGONAUTE
amiRNA	artificial miRNA
ARF	AUXIN RESPONSE FACTOR
ceRNA	competing endogenous RNA
CRISPR	clustered regularly interspaced short palindromic repeats
DCL1	DICER-like1
EDC	N-(3-Dimethylaminopropyl)-N′-ethylcarbodiimide hydrochloride
eRNA	enhancer RNA
FANS	fluorescent-activated nuclei sorting
FACS	fluorescent- activated cells sorting
GFLV	*Grapevine fanleaf virus*
GFP	green fluorescent protein
GUS	glucuronidase
HEN1	HUA ENHANCER1
HYL	HYPONASTY LEAVES1
INTACT	isolation of nuclei tagged in specific cell types
LNA	lock nucleic acid
*MIR*	*MIRNA* gene
miRNA	microRNA
mRNA	messenger RNA
ncRNA	non-coding RNA
NGS	next generation sequencing
PAR	promoter-associated RNA
PHB	PHABULOSA
piRNA	PIWI-interacting RNA
Pol II	DNA-dependent RNA polymerase II
Pre-miRNA	precursor miRNA
Pri-miRNA	primary miRNA
PTGS	post-transcriptional gene silencing
RISC	RNA-induced silencing complex
RNAi	RNA interference
rRNA	ribosomal RNA
SDN	small RNA-degrading nuclease
SE	somatic embryogenesis
siRNA	small interfering RNA
snoRNA	small nucleolar RNA
snRNA	small nuclear RNA
SPL	SQUAMOSA-PROMOTER BINDING PROTEIN-LIKE
sRNA	small RNA
STTM	short tandem target mimic
TF	transcription factor
TIR1	TRANSPORT INHIBITOR1
TM	target mimicry
tRNA	transfer RNA
U	uridine
UTR	untranslated region
YFP	yellow fluorescent protein
ZE	zygotic embryogenesis
